# Chemically crosslinked PVA hydrogels for cartilage substitution

**DOI:** 10.1080/07853890.2021.1896893

**Published:** 2021-09-28

**Authors:** Inês Patacho, Andreia S. Oliveira, Pedro Nolasco, Rogério Colaço, Ana P. Serro

**Affiliations:** a CQE, Instituto Superior Técnico – Universidade de Lisboa, Lisbon, Portugal; b Centro de Investigação Interdisciplinar Egas Moniz (CiiEM), Egas Moniz Cooperativa de Ensino Superior, Caparica, Portugal; c IDMEC and DEM, Instituto Superior Técnico – Universidade de Lisboa, Lisbon, Portugal

## Abstract

**Introduction:**

In recent decades, hydrogels have gained increasing interest as potential cartilage replacement materials. Among these, polyvinyl alcohol (PVA) hydrogels have shown to be very promising candidates due to their biocompatibility, high degree of swelling, and elastic and rubbery nature, which allows them to mimic the natural tissues [1]. The properties of PVA-based hydrogels can be tailored by adding different compounds. The main objective of this study is to investigate the effect of adding two distinct crosslinking agents to the polymeric mixture, on the microstructure, water content, hydrophilicity, and mechanical behaviour of PVA hydrogels.

**Materials and Methods:**

Four types of materials were prepared using a PVA aqueous solution (7.75% w/w) containing two different crosslinking agents: glyoxal at 0.2% and 1%, and glutaraldehyde at 5.95% and 11.9% (where the percentages indicated refer to the mass of the crosslinker relative to that of the PVA). The mixtures were poured into Petri dishes and dried at 37 °C (4 days) and then at 60 °C (2 days). The surface morphology of the samples was analysed by scanning electron microscopy (SEM) after they had been lyophilised for 48 h and coated with an Au/Pd layer. To determine the equilibrium water content (EWC), samples hydrated in pure water were weighed, dried at 60 °C until reached a constant weight, and weighed again. The water contact angles were determined in hydrated samples by the captive bubble method. To evaluate the mechanical properties, compression tests were performed with a texturometer on samples placed in an aqueous medium, using a test speed of 0.1 mm/s until a force of 5 kg was reached.

**Results:**

The results showed that all materials are non-porous and have a similar surface morphology. The hydrogels crosslinked with glyoxal present higher EWC values (69.1% and 69.7% for 0.2% and 1% of glyoxal, respectively), while those with glutaraldehyde have an EWC of 52.8% and 41.5% for 5.95% and 11.9% of glutaraldehyde, correspondingly. Concerning wettability, all samples are hydrophilic and exhibit a water contact angle <55°. The modulus of elasticity of materials prepared with glyoxal is lower (1.1 − 1.4 MPa) than that of those done with glutaraldehyde (6.2 − 9.3 MPa), but their toughness is higher (0.67 − 0.45 MJ/m^3^ *vs* 0.22 − 0.27 MJ/m^3^). For gels crosslinked with glyoxal, the dissipated energy assumes values between 17 − 22% and for samples prepared with glutaraldehyde, it is negligible.

**Discussion and conclusions:**

In conclusion, the nature and amount of crosslinking can determine the properties of PVA hydrogels. Glyoxal-containing materials have a greater water absorption capacity, are less stiff, absorb more energy, and exhibit a lower elastic recovery than glutaraldehyde-containing gels. Overall, glyoxal-crosslinked hydrogels have EWC values and mechanical properties closer to those of the natural cartilage, and therefore should be preferred as potential substitutes of this tissue.
